# Stem Cells as a Target for the Delivery of Active Molecules to Skin by Topical Administration

**DOI:** 10.3390/ijms21062251

**Published:** 2020-03-24

**Authors:** Hamid-Reza Ahmadi-Ashtiani, Parisa Bishe, Anna Baldisserotto, Piergiacomo Buso, Stefano Manfredini, Silvia Vertuani

**Affiliations:** 1 Department of Basic Sciences, Faculty of Pharmacy, Tehran Medical Sciences, Islamic Azad University, Tehran 194193311, Iran; pbishe@iaups.ac.ir; 2 Cosmetic, Hygienic and Detergent Sciences and Technology Research Center, Tehran Medical Sciences, Islamic Azad University, Tehran 19419311, Iran; 3 Department of Life Sciences and Biotechnology, Faculty of Medicine, Pharmacy and Prevention, Master Course in Cosmetic Sciences, University of Ferrara, Via Luigi Borsari 46, 44121 Ferrara, Italy; bsupgc@unife.it (P.B.); smanfred@unife.it (S.M.); vrs@unife.it (S.V.)

**Keywords:** topical, penetration enhancement, delivery, cosmeceutical, drug, skin stem cells

## Abstract

Cutaneous stem cells, gained great attention in the field of regenerative medicine as a potential therapeutic target for the treatment of skin and hair disorders and various types of skin cancers. Cutaneous stem cells play a key role in several processes like the renovation of skin structures in the condition of homeostasis and after injuries, the hair follicle growth and the reconstruction and production of melanocytes. Thus, gaining effective access to skin stem cells for therapeutic interventions that often involve active molecules with non-favorable characteristics for skin absorption is a valuable achievement. The topical route with high patient compliance and several other benefits is gaining increasing importance in basic and applied research. However, the major obstacle for topical drug delivery is the effective barrier provided by skin against penetration of the vast majority of exogenous molecules. The research in this field is focusing more and more on new strategies to circumvent and pass this barrier effectively. In this article the existing approaches are discussed considering physical and chemical methods along with utilization of novel drug delivery systems to enhance penetration of drugs to the skin. In particular, attention has been paid to studies finalized to the delivery of molecules to cutaneous stem cells with the aim of transferring signals, modulating their metabolic program, inducing physiological modifications and stem cell gene therapy.

## 1. Introduction

Skin fulfills essential functions to the animal survival, it is composed of different and organized types of differentiated cells presenting various embryonic origins. Skin provides a fundamental barrier against UV-radiation, temperature, infections, exposure to xenobiotics, pollutants, antigens and trauma. The entire structure needs to be renovated over the entire life and adnexal structures like hair follicles go through growth and regression or rest in a defined cycle. Stem cells can be found in the basal layer of the epidermis, which represents the outer stratum of the skin and in the structures of the hair follicles and sebaceous glands. Stem cells are located in microenvironments called niches that play an important role in their regulation and are responsible for replenishing skin cells both in the condition of homeostasis and after injuries [1,2,3]. Reduction in the number and function of effective stem cell populations results in various types of dysfunctions like chronic wounds and tissue aging [4,5]. On the other side, mutations in stem cell genetic material or any perturbation in the management of stem cell proliferation may represent the starting point of various types of skin cancer [6]. These critical roles made them susceptible targets for therapeutic assets designed to treat human skin and hair disorders such as severe burns and wounds as well as skin cancers and alopecia and the effects of aging [7].

The topical route, seen as a preferential path for transporting active compounds directly to therapeutic targets like stem cells, recently captured the attention of researchers because of the great benefits that it could offer if compared to other approaches. Transferring molecules through the skin performing dermal and transdermal delivery represents a relevant option, it is noninvasive and allows self-administration. These two aspects lead to improved compliance and a considerable reduction in complications and side effects normally associated to injectable drugs making a topical route preferred to parenteral administration where practicable [8,9].

The first and most important obstacle for a topically applied drug is the barrier formed by the layers of the epidermis and, in particular, by stratum corneum, which represents an effective obstruction against the penetration of substances from the external environment. The epidermis only allows passive passage of compounds with molecular weight below 500 Da and adequate lipophilicity. Thus, not many products designed for transdermal administration show during their development the appropriate characteristics to find their way to the people; many new-generation actives such as peptides, proteins and nucleic acids are not compatible with topical administration because of their large molecular size.

Therefore, there is an extreme need to develop, intrinsically safe, permeation enhancement techniques to increase the bioavailability of molecules formulated for dermal and transdermal delivery [9,10,11] and possibly adapt them in view of the new evidence from progressing scientific studies. 

Skin plays a vital role in defending the body against external stimulus of various nature, considering also the fact that skin is the largest and most accessible organ of the integumentary system, it appears to be a promising field for research on stem cells and their role in the maintenance of compartments homeostasis and regeneration [6].

Cutaneous stem cells are found in the epidermal and dermal layers as multipotent follicle, epidermal, melanocyte, dermal sheath, mesenchymal, neural crest, hematopoietic and endothelial stem cells. They recently emerged as an interesting target for healing molecules. Thus, this review concentrates on skin barrier overcoming methods to address readers’ attention on possible applications to epidermis stem cells as a possible target for ingredients of topical formulations that could easily access them and gain interesting effects.

## 2. Skin Stem Cells

Follicular stem cells, epidermal stem cells and melanocyte stem cells (MSC) lie in the epidermis [12,13]. A significant part of the stem cell population resides in a region of the hair follicle known as a hair bulge and in a bunch of cells below the bulge known as the hair germ. It is proven that the hair follicle bulge forms a repository of stem cells in the skin. Stem cells can migrate from this structure to the matrix of the hair follicle, the sebaceous gland and the basal layer in the interfollicular epidermis to give rise to precursors that differentiate into hair cells, gland cells or cells of the epidermal layers [2,13]. The upper segment of the hair follicle is permanent, it consists in the infundibulum that opens the hair canal to the surface of the skin, and in the isthmus. The lower part is transient and undergoes the growth phase (anagen), resorption phase (catagen) and resting phase (telogen). Follicular stem cells take part in the activation of new hair cycles and reconstitute hair follicles [1,14,15].

The basal layer of epidermis is the primary location of mitotically active cells that are able to generate the superior layers structure [16]. Clonal regeneration assays on mouse epidermal cells showed that the stem cells might represent 2–7 percent of the basal layer cells [17]. The terminally differentiated cells in the epidermis are detached from the skin continuously and considering that they lost their division capability, their substitution is dependent on epidermal mitotically active cells [3].

When skin is damaged, follicular and epidermal stem cells collaborate for efficient wound healing. Mitogenic factors stimulate the division of both stem cells and the process of migration from the bulge is accelerated [7,15,16].

Melanocyte stem cells are located in the bulge area and the hair germ. The melanocyte stem cells generate mature melanocytes that produce melanin throughout the anagen phase of the hair growth. Melanin pigments give skin and hairs their colors and dissipate most of the absorbed UV radiation preventing DNA damage and skin cancer [2].

## 3. Stem Cells and Skin Diseases

Over the past two decades, there has been a huge increase in understanding stem cell biology, including skin stem cells. New perspectives in the use of stem cells in the treatment of pathologies and disorders associated with human skin such as skin cancer, alopecia, but also severe burns and wounds, have been provided by extensive research on stem cells and pursuing potential clinical applications.

The important advances made in the field of cell biology have demonstrated the role of stem cells in the skin in the process of repairing it. In fact, stem/progenitor cells of the epidermis play an essential role in the regeneration of skin tissues.

On the light of these experimental evidences, the aims of this section were to highlight the role and involvement of epidermal stem cells in various skin pathologies. 

### 3.1. Skin Disorders and Melanocyte Stem Cells 

#### 3.1.1. Vitiligo 

Vitiligo is an autoimmune disease that arises as a consequence of a variety of genetic and external factors, it affects 0.3%/0.5% of the world population. This pathology causes a progressive loss of skin pigmentation due to a gradual decrease or disappearance of melanocytes.

The interest in vitiligo research has recently focused on skin stem cells, more particularly on stem cells residing in the hair follicle due to their potential in therapies aimed at improving pigmentation. The poor progress made by recent research on the treatment of vitiligo is mainly due to a still poor understanding of the mechanisms underlying the activation of melanocyte stem cells and their proliferation and migration, aimed at repopulating the surrounding basal epidermis. Closely linked to this line of research, there is the study of the most suitable drug delivery methods for the treatment of this cellular population [18].

The treatments available today offer a temporary and often partial remedy. One of the most practiced and effective treatments consists of exposure to different types of UV light, associated with less beneficial topical treatments based on corticosteroids or analogues of vitamin D. 

The pigmentation of hair appeared preserved even in the skin areas affected by vitiligo, a fact that denotes the presence of a reserve of melanocytes attributable to the structures of the hair follicle in the compromised areas.

Repigmentation of the vitiligo lesions occurred due to a variety of patterns, however, a perifollicular distribution of the pigmentation after exposure to UV irradiation was observed. This fact indicates an important involvement of the precursors of the melanocytes residing in the hair follicle. Melanocytic stem cells represent a promising object of study for new research aimed at clarifying their role in repigmentation and at developing therapeutic strategies capable of an effective modulation of their differentiation and consequent migration.

A closer examination of molecular mechanisms underlying the regulation of skin stem cells could lead to the development of drugs capable of treating vitiligo more effectively [19].

#### 3.1.2. Hair Greying 

Hair greying or the loss of production and deposition of pigments in the hair shafts are an evident sign of aging. There are numerous mechanisms, which, acting at different follicular levels and positions, contribute to hair greying: mechanisms such as, for example, defects in stem cells of melanocytes and follicular death of melanocytes. It has been observed that oxidative damage is a key problem common to these processes. In the hair follicle stem cell niche, oxidative stress, accelerated by the depletion of the B-cell lymphoma 2 (BCL-2) gene, leads to selective apoptosis and reduction of melanocyte stem cells, reducing the repopulation of anagen follicles newly formed [20].

Many researchers have speculated that the progressive loss of follicular melanocytes during aging could result from follicular apoptosis of melanocytes and from defects or depletion of melanocyte stem cells.

It has been hypothesized that these processes could be initiated and accelerated by environmental or melanogenic damage to ROS and reduced BCL-2 levels [21]. Nishimura et al. reported a defective maintenance process of melanocyte stem cells during hair greying, which is markedly accelerated with BCL-2 deficiency [22]. Over time, they become dysfunctional, undergo selective apoptosis and consequently fail to repopulate newly formed hair follicles. Age-related graying could therefore act as a protective mechanism by removing defective melanocyte stem cells before they become cancerous. It can therefore be assumed, in the long term, that by modulating only one desired subpopulation of melanocytes or microenvironment would lead to develop new strategies for safe intervention and reversal of greying hair.

#### 3.1.3. Melanoma 

To date, one of the most serious forms of skin cancer with the highest mortality rate is cutaneous malignant melanoma. There are still open hypotheses regarding the identity of the original target cell. Tradition states that, for a long period of time during UV exposure, cutaneous melanocytes progressively accumulate mutations in oncogenes and tumor suppressor genes, which leads to uncontrolled proliferation, acquisition of invasive properties and metastasizing ability [23].

An alternative hypothesis, on the other hand, maintains that melanoma begins in an extrafollicular melanocyte skin stem cell. The mutation induced by UV rays can alter the normally strictly controlled process of self-renewal, expansion and differentiation of MSCs, in addition to their exit from the stem cell compartment. The understanding of these mechanisms of extrafollicular MSCs will provide more indications on the possible mechanisms for the neoplastic development of malignant melanoma [24,25].

If it can be shown that the primary origin of melanoma lies in a damaged MSC or a precursor of the melanocyte, antibody therapy against a specific target will be the best approach to treat and eradicate melanoma, as well as to prevent its appearance. A model was also presented that shows how MSCs are converted into melanoma stem cells following long-term exposure to UVA/UVB radiation on DNA and its repair mechanisms [26].

### 3.2. Hair Loss and Hair Follicle Stem Cells

Different factors such as hereditary, concomitant medical conditions, hormonal, autoimmune disorders, nutritional disorders, environmental factors, psychological factors and aging cause hair loss. Harmful factors affect the hair cycle by decreasing the activity of stem cells and the regeneration capacity of the hair follicle. Alopecia is commonly considered a defect with apparently no significant health consequences. However, one should not underestimate the fact that hair loss can affect self-acceptance, causing depression and anxiety [27,28]. Additionally, an early onset of androgenic alopecia is associated with both an increased metabolic syndrome and an increased risk of ischemic heart disease [29]. The ubiquity of alopecia encourages research into new, more effective therapies aimed at the regeneration and neoregeneration of the hair follicle.

Several studies have shown that both human hair follicle stem cells (HFSC) and dermal papilla cells (DPC) undergo changes following alopecia [30,31]. These two types of stem cells ensure the conditions for proper hair regeneration. In the case of cicatricial alopecia (lupus erythematosus and lichen planus), inflammatory cell infiltration around the swelling is observed, which causes an irreversible loss of HFSC. In irregular and androgenic alopecia, although progenitor cells are damaged, HFSC are preserved, which is why this type of alopecia can be reversible [31]. Significant improvements in intraoperative stem cell approaches, from in vivo models to clinical investigations are reported in the literature, which report the potential tools and regenerative functions of various cell populations in the hair regrowth process. Mesenchyme signals derived from stem cells and growth factors obtained from platelets influence hair growth through cell proliferation to prolong the anagen phase (FGF-7), induce cell growth (ERK activation), stimulate hair follicle development (-catenin) and suppress apoptotic signals (release of Bcl-2 and activation of Akt) [32]. Another aspect not to be underestimated is hair loss following chemotherapy, which causes both psychosocial stress but also rejection of chemotherapy itself. A recent study reported that priming mobilization of hair follicle stem cells causes a loss of their resistance to DNA damage, resulting in permanent loss of regeneration after alkylating chemotherapy [33].

### 3.3. Psoriasis

Epidermal stem cells are involved in different types of skin diseases. Further research work is needed to clarify the precise role of these cells in the pathological process, in this field there is scientific evidence of the involvement of epidermal stem cells in psoriasis. The role of epidermal stem cells (ESC) in disorders associated to psoriasis is currently not fully clarified, studies regarding this aspect require specific attention due to the fact that ESC are precursors of skin cells and structures involved in the development of psoriasis like keratinocytes. Considering the studies collected to date, several kinds of stem cells/progenitor cells appear to be involved in a multisystem disease like cutaneous psoriasis [34].

In a study by Gago-Lopez et al. [35] the authors showed the reduced expression of c-JUN and JUNB in hair follicle stem cells in patients with scalp psoriasis proving the fundamental role of mutations in stem cells in the development of the disease.

Recent studies highlighted potential therapeutic uses of stem cells of different origins in the treatment of psoriasis, even if the underlying mechanisms remaining are not clear and are elusive. For example, a recent clinical communication describes the positive effects of mesenchymal stem cells infusions, capable of decreasing the severity and extension of psoriasis. It is assumed that the mechanisms at the basis of this activity are the downregulation of the expression of proinflammatory cytokines and the inhibition of the infiltration of cells involved in the immune response to the structures of the skin [36].

### 3.4. Junctional Epidermolysis Bullosa

Junctional epidermolysis bullosa (JEB) is a serious genetic disease triggered by mutations in genes encoding the component of the dermal–epidermal junction laminin-332. Surviving patients with JEB present severe chronic wounds to large areas of skin. Hirsch et al. [37] assessed the validity of therapies including stem cells in the treatment of this severe disease, more in particular a treatment involving transgenic epidermal stem cells was able to restore an entire functional epidermis on a seven-year-old patient suffering from JEB, without adverse events after 21 months follow up.

### 3.5. Basal Cell Carcinoma

Recent studies demonstrated that mutations in somatic stem cells might have a role in the development of various types of tumors [38]. It is assumed that a common cancer like basal cell carcinoma can originate from skin stem cells of follicular or interfollicular origin, even if the fact that there is a strong differentiation between somatic stem cells and cancer stem cells must be underlined. It is possible that during the process of carcinogenesis, cells, which do not have stem cell characteristics, may obtain them by becoming a potential clonal originator of a tumor. Therefore, the existence of tumor stem cells in neoplasms does not necessarily mean that the tumor originates from a somatic stem cell. In this context it is necessary to deepen the knowledge regarding cancer stem cells detection (specific markers like Cytokeratin 19, transcription factor p63, Integrins and Bmi-1) and selective targeting in order to develop more focused therapeutic assets [38].

### 3.6. Squamous Skin Carcinoma

Moreover, dealing with another important type of skin cancer called squamous skin carcinoma, genetic mutations in stem cells located in the bulge area seem to be able to lead to a more aggressive carcinoma than the same genetic alterations occurring in other cell populations of human skin [39]. Additionally, in this case further studies are needed to develop new therapeutic strategies able to target selectively cancer stem cells responsible for metastasis or their specific functions rather than normal epidermal stem cells.

## 4. Advantages of Topical Administration 

In recent years, many innovative therapies involving stem cells have been developed or are being tested. They mostly exploit stem cells of various origins (mainly mesenchymal) seen as tools aimed at tissue regeneration in different contexts. These particular cells have potential uses in the field of regenerative medicine, which concerns both surgical and aesthetic aspects. It should be emphasized that stem cells have so far been considered more as a therapeutic tool than as a target for focused therapies. In this context, it is essential to summarize strategies able to allow potential therapeutic approaches to stem cells from a topical point of view, considering in particular the skin stem cells as an important site of action for various types of therapies able to influence their metabolism and differentiation with the aim of solving important skin pathologies and aesthetic problems. The analysis of the potential therapeutic approaches to skin stem cells available from the topical point of view and related issues can be considered as an important contribution to the conception stage and development of research projects in this direction.

The future of the study of skin stem cells is ultimately their in vivo regulation by means of targeted innovative therapies involving topical drug delivery strategies.

As mentioned above, the choice of the topical route to administer drugs whose therapeutic target is stem cells, can be currently considered a preferential approach compared to others, because of the numerous benefits it could offer and which are the subject of this paragraph [10,40,41]:

1. When using oral route or systemic injection, plenty of cells will be affected and only a small portion of the active molecules actually reaches the target organ, the skin, unless the therapeutic molecule or its vehicle is designed to only interact with specific cells in a particular area. Moreover, the oral route, due to rapid degradation and limited absorption, cannot deliver most therapeutic peptides and proteins. Additionally, many drugs are affected by first hepatic metabolism and diverse conditions of absorption in the gastrointestinal tract like changes in the acidity degree, presence of enzymes or gastric emptying time.
2. In the case of local injection therapy, complications related to production of injectable drugs, risks of administration and poor patient compliance are the negative points, which propel us to use the topical method.
3. Topical administration provides the most accessible route for targeted delivery of molecules to a specific skin area.
4. Patients adhere well to medication because it is a painless and noninvasive technique and provides the convenience of application and compatibility for self-medication.
5. Skin makes a vast area available for the administration of drugs.
6. Systemic side effects are reduced.
7. Controlled and prolonged drug delivery can be accomplished and fluctuation in drug levels is avoided as a consequence.
8. Consumption of drugs with a narrow therapeutic index or short biological half-life is possible by this route because the minimal required dose of the drug is delivered directly to or near the intended site of action.
9. Efficacy is elevated with the lower total daily dose needed by continuous drug input.
10. The medication can be easily stopped, when needed.

Rationally, conventional drug delivery routes have many inherent limitations that could potentially be overcome by advanced drug delivery methodologies such as transdermal delivery.

## 5. Pathways of Penetration

**Scheme 1:**
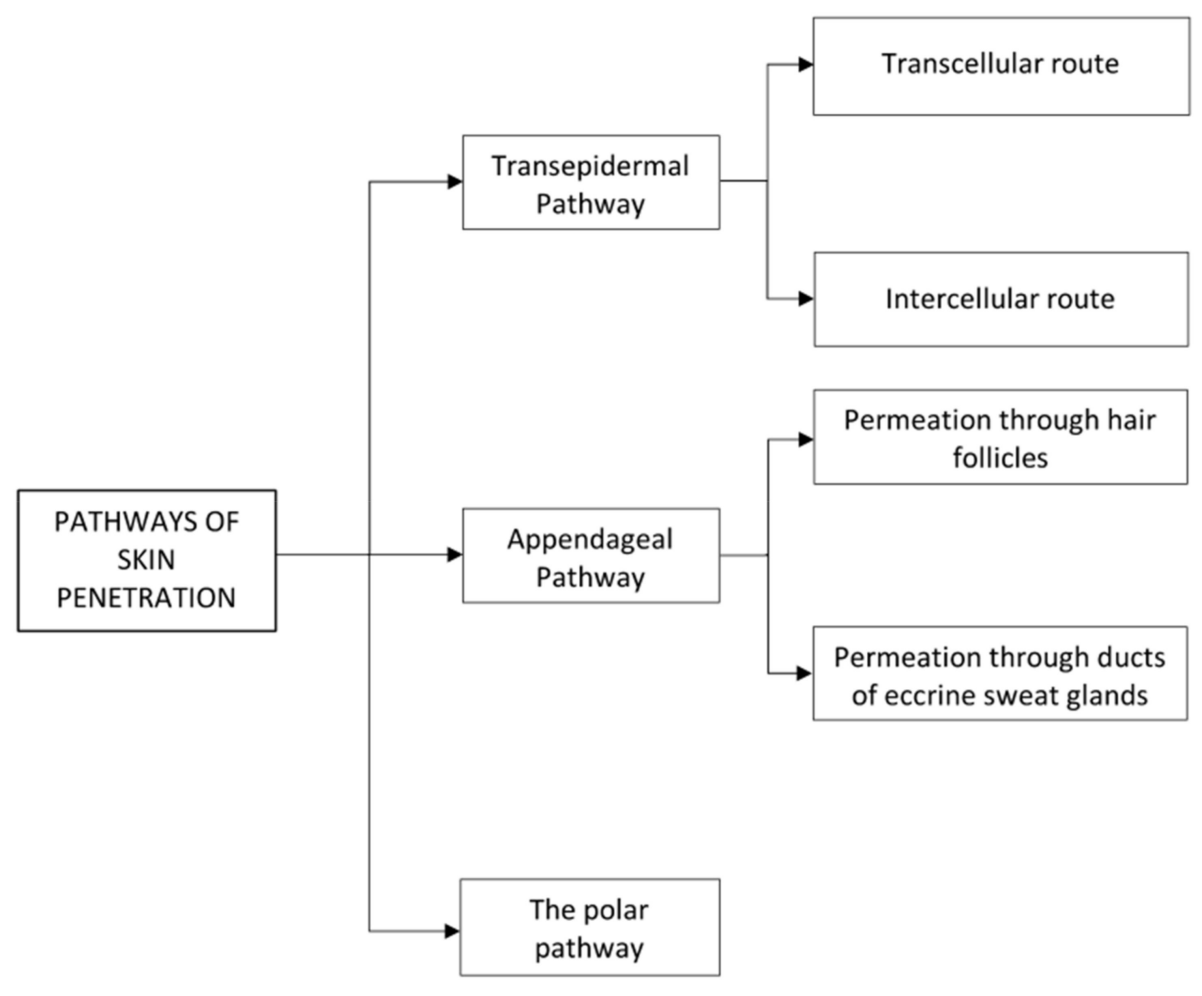
Pathways of skin penetration.

**Figure 1:**
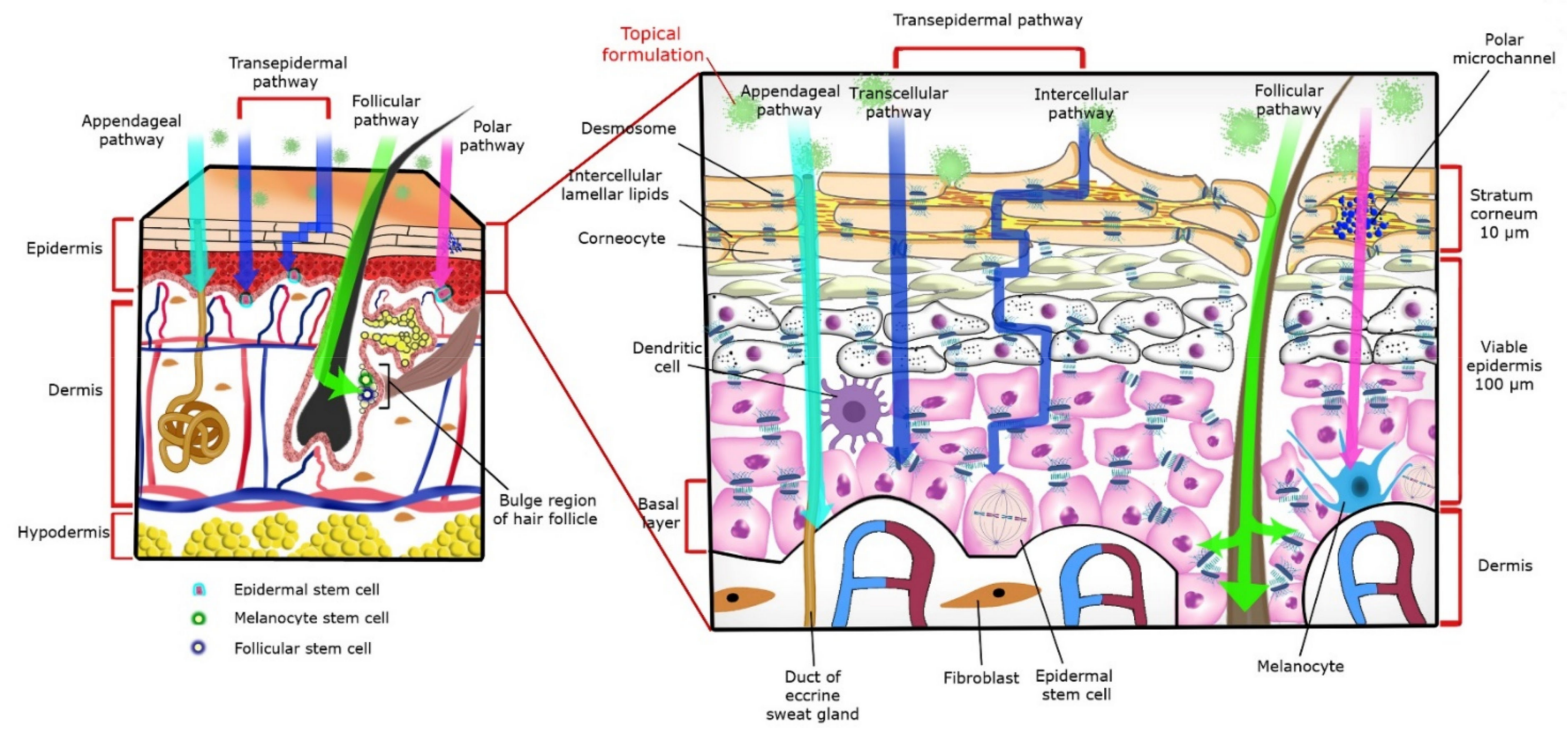
Pathways of penetration to the skin.

A molecule can penetrate into the skin through different pathways (Scheme 1) including the transepidermal route (diffusing across the epidermis), the appendageal pathway (through hair follicles or sweat ducts) or polar pathway (through polar microchannels) as illustrated in Figure 1 [42]. 

The cutaneous penetration pathways through the stratum corneum via intercellular/intracellular routes, and through the appendageal pathway result in topical and transdermal delivery. The choice to treat skin diseases such as psoriasis, contact dermatitis and skin cancer with locally applied therapies is a promising approach as the drugs are directly administered in the skin layers.

The transcellular pathway requires that the molecule cross the intermittent layers of cells and extracellular matrix: this however implies a series of partitioning and spreading in hydrophilic and lipophilic domains, which is undesirable for most drugs. Since the internal environment of cells is generally more hydrophilic than the external one, the transcellular pathway will be favored for hydrophilic molecules, while the intercellular pathway for lipophilic ones [43,44]. The transepidermal pathway seems to be the ideal path of penetration for the molecules to reach the stem cells in the basal layer of the epidermis.

The appendageal or shunt pathway encompasses permeation through hair follicles (the follicular route) or ducts of eccrine sweat glands. The content of the eccrine sweat glands is mainly hydrophilic, while a certain degree of lipophilicity for compounds entering the hair follicle might be necessary mainly due to the sebum emission into the orifice of the follicular duct. The appendages form incoherence in the stratum corneum that are relatively effortless pathways of penetration through the stratum corneum. In the past, the appendageal route has long been considered of minor importance, since the apertures account for only 0.1% of the total skin surface. However, the follicular route has gained significant research interest in recent years. In the upper section of the hair follicle (the infundibulum), thinned stratum corneum provides a weaker barrier displaying an area of additional absorption. Furthermore, local administration of drugs to the skin with the aim of high accumulation in the follicular region performs prolonged drug delivery. The other reason that draws great attention to this route is the presence of attractive regions in the follicle to be targeted for topical delivery such are the sebaceous gland and the bulge of the hair follicle, which hosts the stem cells. The multipotent, masterly proliferative and easily accessible stem cells located in the bulge region are to be integrated in the therapeutic targets of cutaneous regenerative medicine or gene correction of inherited hair disorders and also because that various forms of skin cancer have been thought to originate from this region [43,45,46].

## 6. Barriers to the Topical Route of Administration

The skin acts as an effective barrier that prevents and regulates the entry and exit of many substances, it also provides effective protection from excessive water loss thus maintaining the homeostasis of the organism. The amount of water that vaporizes from the skin surface, known as transepidermal water loss (TEWL), can be used as measure of the skin’s protective barrier status. The patients with dry skin and atopic dermatitis, who have a disturbed skin barrier have shown a rise in TEWL. It is well known that the barrier function of the skin is performed mainly by stratum corneum and that it is the most important layer in restraining the loss of water [8,47]. 

Although the stratum corneum is distinguished as the most important physical barrier, further investigations revealed that also deeper layers of epidermis are essential for barrier function. By using calibrated microdermabrasion to selectively eradicate the stratum corneum or full epidermis, it was discovered that removal of the stratum corneum increased skin permeability markedly. However, abrasion of the full epidermis added skin permeability by another 1–2 orders of magnitude [48,49].

The stratum corneum is composed of 10–15 layers of nonliving cells filled with keratin filaments in a cement of nonpolar lipids. Diverse factors cooperate in this external layer of the skin to create an effective barrier against material transit. Corneocytes are closely connected to each other by corneodesmosomes, which are fundamental to maintain the layer integrity. Below the cytoplasmic membrane, corneocytes have a cornified envelope with a strong protein lipid polymer structure that effectively avoids the entrance of substances to the intracellular environment. Lipids of the intercellular matrix are organized as lamellar layers and form a continuous domain in the stratum corneum. Ceramides, free fatty acids and cholesterol are three major elements of the extracellular matrix. Several experiments revealed that extraction of intercellular lipids or disorganizing their lamellar formation by chemical agents have a fatal impact on the barrier function of the stratum corneum. It is supposed that the long-chain lengths and firm packing of ceramides and fatty acids are the determining factors promoting physical stability of lipid bilayers. Moreover, it is important to mention cholesterol sulphate, which, constituting up to 2%–5% of the total lipids weight, appears to assist the formation of the lipid lamellae and stabilizes the stratum corneum by inhibiting enzymatic degradation of corneodesmosomes [43,48].

The nucleated epidermal layers are also contributors in the barrier function through tight, gap and adherens junctions, as well as through desmosomes and cytoskeletal elements. Tight junctions are joints that connect adjacent cells, bounding the passage of molecules in the paracellular space. Tight junction proteins and analogous structures are found in the interfollicular epidermis as well as in the skin appendages in human skin. Tight junctions might be a rescue system when stratum corneum barrier is challenged or damaged. The contribution of gap junctions in the barrier function of the epidermis was studied in vivo on mice with a defect in the structure of the channel-forming proteins evidencing an imperfect barrier behavior. A perturbation in the barrier function has also been declared after alteration of the adherents junctions and desmosomes [48].

## 7. Strategies to Enhance Skin Penetration

As seen in the previous sections, there are different ways of penetration through the skin, which in addition to providing protection for the body acts also as a selective barrier. These strategies can be seen as tools to gain access to the different types of skin stem cells and their surrounding microenvironment for different purposes. Regarding this, various strategies can be applied to promote the penetration of drugs to the skin (Table 1). The simplest approach is to remove the stratum corneum or physically bypass it, by creating both macroscopic pathways and micropores through which molecules can penetrate. Chemical penetration enhancers and drug delivery systems temporarily alter the barrier properties of the skin by interacting with the stratum corneum components through different mechanisms or optimize partition or diffusion characteristics of the drug [9].

Different strategies have been studied in recent decades that we can classify in the passive and active penetration enhancement methods. The methods of improving passive penetration involve optimizing the formulation and the carriers of drugs to increase the flow. These methods are not sufficient for polar and high molecular weight molecules, such as plasmid DNA, peptides and vaccines. The methods of active improvement of skin penetration, on the other hand, mainly involve the following mechanisms: abrasion of the stratum corneum [50,51,52,53], electroporation to generate transient aqueous pores in the lipid bilayers of the stratum corneum [54], ultrasound waves to interrupt lipid packaging in the stratum corneum [55,56] and the use of microneedle arrays to bypass the stratum corneum [54]. Here following a brief discussion of the enhancement methods that most involve skin stem cells as the final target for skin penetration.

### 7.1. Physical Enhancement

#### Microneedling 

It is a technique of piercing the stratum corneum by making use of microneedles that are 10-200 μm in height and 10–15 μm in width arranged on a small patch, thus creating micron size pathways that direct the hydrophilic high molecular weight compounds straight to the epidermis. The various types of microneedles include solid, coated, dissolving, hollow and hydrogel-forming microneedles that can be obtained from diverse materials like silicon, metals, ceramic, polymers, carbohydrates and silica glass. They are designed to overcome the biologic barrier of the skin whilst not reaching the nerve ends to be painless. This system has spread its application to many fields like oligonucleotide delivery, vaccine delivery and even in cosmetics. Microneedling is a way to revitalize the skin without destroying the epidermis [54,57,58]. 

### 7.2. Polymeric Nanoparticles-Based Delivery Systems for Topical Application

Nanosized carriers, such as liposomes, ethosomes, solid lipid nanoparticles and nanoemulsions, have been widely applied as topical formulations to enhance cutaneous delivery [59,60].

#### 7.2.1. Liposomes

The liposomes are prepared vesicles structured as lipid bilayers enveloping an aqueous core that are frequently used as vehicles for delivery of substances in a controlled manner to particular areas of skin or its layers. According to their special structure, liposomes can carry hydrophilic agents in their internal aqueous partition and lipophilic molecules via the nonpolar tails of the bilayer section. Liposomes, with their small size and resemblance to the skin structure considering their lipid composition, provide consistent facilitation to the penetration inside the horny layer that is more pronounced if compared to conventional dosage forms. In the last years, particulates such as liposomes have drawn great attention as a result of their effectiveness to improve penetration into the hair follicle structures. In order to consent to particles to penetrate deep into the hair follicles, they must have a size between 400 and 700 nm to be considered optimal. On the contrary, particles larger than those indicated above will remain on the surface of the skin, while those of smaller dimensions will reach a significantly greater depth [46,57,61].

#### 7.2.2. Ethosomes

Ethosomes, a novel type of liposomes containing 20%–50% of ethanol (*v*/*v*), are so called for the presence of ethanol. Ethosomes are deformable vesicular carriers, mainly made of phospholipids, and have the potential to achieve these therapeutic requirements because they can increase drug loading by increasing solubility and transdermal absorption. This innovative vesicular system has been used in recent years for the transdermal delivery of drugs as it allows them to pass through the skin and its accumulation.

Ethosomes have an aqueous core, which contains the ethanolic solution of the drug and the outer layer comprises of the lipid bilayer. The effect of ethanol fluidizing the bilayers of phospholipids contributes to the creation of vesicles with a malleable structure, which enable it to attain molecules (drugs, pharmaceuticals or active agents) to deeper layers of the skin [62,63].

The interaction of a drug, encapsulated in nanosystems, with the skin no longer depends on the drug but on the formulation. If the size of these nanosystems is kept below 300 nm, the limits of percutaneous administration of the drug are exceeded as these systems can be transported to the deeper layers of the skin [64]. Ethosomes can be potential carriers for transportation of drugs across the scalp for treating androgenic alopecia and male pattern hair loss [65].

#### 7.2.3. Solid Lipid Nanoparticles

A solid lipid nanoparticle (SLN) is a sphere-shaped nanocarrier with an average diameter between 10 and 1000 nm. These preparations were developed at the beginning of the nineties starting from o/w emulsions and replacing the liquid lipid constituting the oil phase with a lipid component solid at body temperature. SLNs hold a solid nucleus of physiologically compatible lipids that can solubilize lipophilic molecules. Different emulsifiers can stabilize the lipid core. SLNs have shown interesting capacities as topical drug transporters.

The manufacturing process is very important and influences delivery, and both a quick and slow release of the drug from SLN can be achieved: the cosmetic or pharmaceutical active agent is dissolved in the melted lipid phase, which is dispersed by high speed stirring in an aqueous surfactant solution heated at the same temperature. The obtained mixture is homogenized in high pressure conditions producing an o/w nanoemulsion. The emulsion droplets crystallize after cooling yielding lipid nanoparticles presenting a solid matrix.

Superficial positioning of the drug in the structure of the lipid particles provides a high concentration of active compounds in the skin immediately after application. Whereas, dispersion of the drug inside the core of SLNs creates a drug storage that allows controlled drug release. An excess of surfactant molecules in the formulation containing SLNs may additionally act as a penetration enhancer. SLNs applied to the skin may either penetrate the stratum corneum or preferably enter and gather in skin appendages permitting the contact between active compounds and stem cells [66].

#### 7.2.4. Nanoemulsions

Nanoemulsions (NE) are stable colloidal systems made up of an oil phase, an aqueous phase and a mixture of surfactants and cosurfactants acting as stabilizers, with droplets diameter smaller than 100 nm. Nanoemulsions are transparent dispersions of nanoscale droplets very similar to microemulsions but obtained by different methods. Nanoemulsions are defined as thermodynamically unstable systems produced by mechanical shear in contrast to microemulsions that are thermodynamically stable and form spontaneously [67].

These nanosized delivery systems proffer considerable prospects for targeted drug delivery to and via the skin. They can enhance the skin penetration of both small and macromolecules that do not permeate the stratum corneum in sufficient magnitudes to provide a therapeutic outcome. This has been attributed either to the action of their components on the skin, to their phase structure and to their particle size. These nanosystems offer significant superiority if compared to gross emulsions and gels, in the formulation and vehiculation of hydrophobic molecules by improving their solubility and bioavailability. Moreover, various examples of nanosystems designed to enhance transfer of hydrophilic substances have been reported. NEs not only increase the permeation in the stratum corneum, but also represent the ideal delivery into the hair follicles structures. Considering the presence of sebum in hair follicles, it is expected that vehicles such as NE with the presence of an oil phase, surfactant and alcohol could facilitate intrafollicular delivery of both hydrophilic and hydrophobic active ingredients. Relative simplicity and cost-effectiveness of the manufacturing processes are the other benefits of these systems [68]. Several studies report the presence of nanoparticles in hair follicles following local application, indicating that direct application of active ingredients may be the optimal choice for treating hair growth and hair loss [69,70,71].

In addition to this aspect, the presence of nanoparticles in the hair follicles can also favor the release of active ingredients capable of manipulating the proliferation and differentiation of the hair follicle stem cells. Potential applications could be directed to the treatment of dermatological conditions and wound healing.

#### 7.2.5. Nanocrystals

Drug nanocrystals are nanoparticles of pure drug in a crystalline state with no carrier material. They are stabilized by polymeric or surfactant-based stabilizers and have a particle size between 1 and 1000 nm. The advent of nanoparticles in the form of nanocrystals led to the evolution in the effective delivery of pharmaceutical and cosmetic active agents. The nanocrystals have characteristics that make them safer for topical application than other nano ingredients-based formulations: they have a greater load capacity and require a lower amount of surfactants to be stabilized. Drug nanocrystals can also be used for all those poorly soluble drugs that need a better profile of solubility and bioavailability. Nanocrystal based formulations have shown increased cutaneous penetration. Due to increased saturation solubility of the drug nanocrystals, it creates a concentration gradient between the formulation and the target cells in the skin, which results in raised absorption of the drug. The use of formulations based on nanocrystals allows a prolonged and continuous release of drugs that exploit the appendage route to reach the target site. Furthermore, the accumulation of drug nanocrystals in the hair follicles, through the creation of a deposit of suspended nanocrystals, contributes to a gradual increase in penetration into the surrounding skin layers of the drug. Ease of manufacturing and enhanced stability fascinate researchers and open up scenarios for future development of nanocrystal based topical formulations [72].

### 7.3. Combining Delivery Approaches

A strategy that can be useful to increase dermal delivery is to exploit the combination of different approaches. In some cases synergistic systems employing combinations of different penetration enhancers operating through different mechanisms, are more efficient in overcoming the protective barrier of the skin if compared to single methods from both the point of view of the extent of absorption and of the efficacy and safety of the drug [73,74].

Several studies [75,76] have confirmed the accumulation of nanoparticles applied locally on the surface of the stratum corneum or in hair follicles: these evidences demonstrate that the combination approach of nanoparticles and microneedle arrays could potentially facilitate deposition or the permeation of nanoparticles into the deeper layers of the skin. It has therefore been shown that it is possible to use microneedles to improve the delivery of drug-loaded nanoparticles to the deeper layers of the skin where most dermatological diseases originate. The combination of microneedles and targeted nanoparticle techniques may also provide an alternative route for delivering drugs into specific cells/tissues.

Another example of an approach to transdermal delivery that uses multiple synergistic tools is SPACE-Peptide, a cell penetrating peptide able to deliver various types of therapeutic active molecules into skin after direct chemical conjugation. The conjugation process requires chemical modification of the cargo, which can result in a substantial change in its properties. In addition, conjugation may not be possible for certain drugs, thus limiting the applicability of the peptide as an enhancer. Including SPACE-peptide in a lipid-based carrier system, able to encapsulate and deliver macromolecular actives avoiding chemical conjugation, can circumvent this restriction. 

The combination of different delivery enhancement tools allows the SPACE-ethosomal system to significantly boost the transport of hydrophilic macromolecules into the skin superficial structures, with the highest concentration in the epidermis and minimal penetration to the dermis and systemic circulation. The effective penetration enhancement of this formulation originates from the synergistic action of SPACE-peptide, ethanol and lipid vesicles [77].

## 8. Aims of Accessing Skin Stem Cells

Stem cells, due to their great capacity to perpetuate their undifferentiated state and, at the same time, to generate differentiated progeny, are at the centre of many research projects in regenerative medicine. This therapeutic field seems to offer promising opportunities for the treatment of currently incurable diseases. The predominant strategy adopted so far in regenerative medicine is to treat patients through the transplantation of exogenous stem cells, or their differentiated derivatives, directly at the target site. A complementary alternative to cell transplantation is to regulate stem cells in vivo, for example, by renewing them or accelerating their proliferation so that the tissue is restored or positively influenced by the patient’s cells without the need to remove the tissue beforehand, to cultivate and preserve the cells ex vivo and to perform grafts. Topical delivery strategies find potential uses also in the modulation of the microenvironment surrounding cutaneous stem cells known as the niche, which plays a fundamental role in the management of the correct renewal and turnover of the skin structures and skin appendages operated by the stem cells. More specifically, the aims of accessing skin stem cells can be divided into signaling, metabolic programming and gene therapy approach.

### 8.1. Signaling

Proliferation of cutaneous stem cells is physiologically controlled by various types of signals, e.g., growth factors, received from the microenvironment where they are located consisting in stem cell niches, epidermal cells overlaying the basal layer stem cells, cells within the dermis and dermal papilla at the bottom of each hair follicle and skin adipocytes acting from a relatively longer distance [6,78]. Growth factor signaling is a cell signaling pathway that regulates the growth and evolvement of an organism. Secreted growth factors from the niche bind to stem cells transmembrane receptors triggering cell signaling cascades that promote proliferation and differentiation [79].

Emitted signals such as fibroblast growth factor-7 (FGF-7), FGF-10, insulin-like growth factor (IGF), epidermal growth factor (EGF) and transforming growth factor-α (TGF-α) from dermal fibroblasts play a key role in proliferation of basal epidermal cells. Transition from the basal to the spinous layer requires Notch signaling, a highly conserved pathway involved in a wide variety of developmental processes.

In contrast to stem cells residing in the epidermis, a structure that regenerates incessantly, hair follicle stem cells are maintained in the dormant state during the majority of the hair cycle and only multiply in the anagen phase. Several factors secreted by dermal cells and stem cell progeny take part in the regulation of the proliferative status of the bulge region and hair germ stem cells. The dermal papilla specific signals are also essential in the initial phase of hair regeneration. During the early telogen phase the levels of hair germ-stimulating factors including FGF-7, FGF-10, TGF-β2 and the bone morphogenetic protein (BMP) inhibitor noggin, rise in the dermal papillae. Furthermore, adipocyte progenitor cells secrete platelet-derived growth factor-α (PDGF-α) to activate PDGF signaling in dermal papillae, which afterward activates the hair germ. Wingless/integrated (WNT) signaling, a pathway regulating important aspects regarding cell fate determination and cell migration also during embryonic development, is fundamental for hair germ activation. A recent study shows that bulge stem cells activation depends on sonic hedgehog (SHH), a potent mitogen secreted by the newly formed transit-amplifying cells matrix.

Moreover, signals from both hair follicle stem cells and dermal papillae are essential to synchronize melanocyte stem cell activation and differentiation.

In skin and hair disorders, signaling directed to stem cells deviate from the normal state. As we age, hair follicle stem cells become increasingly resistant to activation due to more BMP inhibitory cues and/or fewer WNT-activating signals [2]. 

In case of injury, the healing process is regulated by a collection of growth factors and cytokines able to reinforce proliferation of epidermal cells. Nevertheless, a deficiency of cytokines and growth factors caused by elevated levels of inflammatory cells that secrete proteases like matrix metalloproteinases makes the chronic wound’s healing process difficult and hesitant [79,80]. The absence of regenerative signals impacts the process of hair follicle morphogenesis leading to scar formation. It was demonstrated that activation of the SHH pathway reinstalls dermal papilla, which is required and sufficient for hair follicle neogenesis. Thus, hair follicle stem cells activity was promoted and the fibrosis was prevented [81]. 

Another example of regenerative medicine application involving signaling processes is a repigmentation treatment against vitiligo outcomes. Vitiligo is characterized by progressive autoimmune destruction of melanocytes. Observations revealed that the melanocyte stem cells in the hair follicle remain intact in patients affected by vitiligo. Durable repigmentation is obtainable by designing pharmacological compounds that can specifically activate melanocyte precursors [19].

Moreover, considering UV-mediated carcinogenesis, signaling modulations play an important role. In humans, excessive exposure to UV radiation increases the risk of oncogenic transformation of long-lived skin stem cells. Tumor-initiating cells, frequently referred to as cancer stem cells, originated from stem cells precursors are also influenced by their niches. Basal cell carcinoma, the most common cancer worldwide, is rooted in deregulated, sustained SHH signaling. It is possible to suppress tumor formation and progression using active compounds able to control the regulatory signals involved [2].

### 8.2. Metabolic Programming

Novel studies about stem cells self-renewal and lineage choice have indicated that, along with growth factors, a variety of metabolic pathways also play a role in the regulation of stem cells fate. Metabolic modulation can determine the dormancy or proliferation of adult stem cells. Therefore, attempting to modulate involved metabolic pathways can be a useful approach in the field of regenerative medicine and prevention of aging [82]. For example, observation of metabolism in hair follicle stem cells has shown that, unlike other cells in the epidermis, pyruvate produced in the glycolysis in these cells is converted more to lactate instead of entering the mitochondria, and it seems that lactate generation is critical for triggering hair cycle. Moreover, the inhibition of lactate dehydrogenase induces hair follicle stem cells to remain quiescent.

In certain cases, hair follicles fail to activate, which is a possible reason of hair loss. Utilizing small molecules that are able to increase lactate biosynthesis in hair follicle stem cells can topically stimulate the hair cycle. One of these molecules, called RCGD423, activates the JAK-Stat pathway, which leads to the increased production of lactate and subsequently stimulates hair follicle stem cell activation and quicker hair growth. The other active compound, called UK5099, blocks pyruvate from entering the citric acid cycle, which drives the production of lactate in the hair follicle stem cells and accelerates hair growth in mice. The ability of small molecules of reinforcing hair follicle stem cells activation could be useful not only for anti hair loss treatments, but also for wound healing innovative treatments in the field of regenerative medicine. Intense efforts are ongoing and advisable in basic and applied research concerning this field [83].

### 8.3. Gene Therapy

Postnatal stem cells with both self-renewing and differentiative capacity, provide cell replacement in tissues and organs throughout the whole life. They are thus the obvious targets of both long-term and transient regenerative treatments and anticancer gene therapies [84]. The highly proliferative stem cells of the bulge region, cyclically regenerate the lower follicle and in the case of wounding they contribute to restoring the epidermis. Hence, the topical delivery of transgenes to hair follicles has great potentialities in the future development of treatments regarding disorders of the hair and skin. The primary challenge of stem cell gene therapy is developing methods to transfer and target DNA and si-RNA molecules to the stem cells of the bulge area, besides protecting them from degradation. For this purpose, particles are valuable carriers [14,85]. In a study by Li and Hoffman (1995) [86], it is reported that topical administration of the lacZ reporter gene trapped in liposomes resulted in specific targeting with gene transfer to the hair follicle matrix cells and follicle stem cells in mice, whereas topical application of the naked lacZ gene did not lead to targeted gene transfer. Except for follicular cells, no other cells in the epidermis or dermis were transfected with the reporter gene entrapped in liposomes. This selectivity of gene delivery by topical liposome application suggests the practicability of targeting hair matrix cells and follicle stem cells for various therapeutic purposes, for example to restore hair color perhaps by transference and expression of the tyrosinase gene and to restore hair growth in alopecia by transference of proper genes [86].

Use of transitory gene therapies is rising as important methods for the supply of growth factors required in the specific case of wound healing treatment. Clinical application of topical growth factors, despite the encouraging results, is constrained by several negative factors such as their short half-lives, their inactivation by proteases in the wound bed, their low delivery and bioavailability from the utilized delivery systems and the consequent need of high initial doses and frequent applications. The combination between growth factor therapy and gene therapy led to the individuation and development of genes coding for the production of growth factors usable for acute and chronic wound healing treatments. Successful delivery of growth factor genes to the wounded skin requires well-designed delivery systems to ensure an effective transfection [87,88,89].

In work by Dou et al. (2007) [82] hair follicles and epidermal cells were targeted by keratinocyte growth factor-1 (KGF-1) DNA plasmid. Their findings indicated that topically applied KGF-1 DNA plasmid could improve epithelial thickness and strength, illustrating the potential of this approach to restore compromised skin. For improving transfection efficiency of topically delivered DNA plasmid, microdermabrasion has been effectively used to increase skin permeability [90].

Another target of gene therapy are cancer stem cells, seen as a distinct subset of the cancer cell population able to generate tumorigenesis and metastasis that are the driving force of a vast majority of skin cancer types. Genetic or epigenetic alterations in tissue stem cells or in some of their differentiated derivatives can lead to uncontrolled self-renewal and tumorigenesis [84]. If a genetic lesion occurs at the level of the oligopotent progenitor cells in the hair follicle, tumors of various epidermal cell types can occur and if the lesion is at the level of the bipotent basal cells, a squamous or basal cell carcinoma might result. Successful cancer treatment must target both the non-cycling stem cells and the dividing cancer cells. This may be achievable if specific stem cell signals leading to proliferation or activation of gene expression that blocks apoptosis are knocked down using gene therapy. With progresses in innovative approaches utilizing specific inhibitory RNAs, such as the kind of therapies may now be possible but critical difficulties in delivering the inhibitory active in a targeted manner to the cancer stem cells have yet to be developed. Liposomal carriers and virus-based expression vectors may be used as delivery systems to provide sufficient concentrations of siRNAs in the target site [91].

## 9. Conclusions

In this review the state of the art of various strategies concerning topical delivery of active compounds to skin structures have been taken into consideration, described and discussed in view of their potential interest in the targeting of stem cells.

Indeed, with the expansion of scientific knowledge concerning the anatomy, physiology and pathological aspects of the skin and the evolution of technological capabilities, efficient topical drug delivery systems and formulations have, in some cases, succeeded in the challenging assignment of overcoming the tight barrier constituted by the stratum corneum of the epidermis. This achievement allows the access of active compounds of various sizes and characteristics, including small molecules and large biomolecules, which would not be able to find access without a suitable vehicle, to underlying layers of the skin and the release of the active directly to the therapeutic target.

About this, physical and chemical methods, novel carriers and synergistic methods have been discussed in this work.

Over the past two decades, our comprehension of the association of skin stem cells with their surrounding microenvironment, known as the niche, has increased significantly. Studies have shown that in many skin and hair disorders, the state of the microenvironment capable of regulating and influencing stem cells results altered. Therefore, developing the capability of manipulating and rehabilitating the stem cells microenvironment may open up new scenarios and therapeutic assets usable in the case of skin and skin appendages problems for both pharmaceutical and cosmetic purposes. The fields of application of this knowledge reside in regenerative medicine, cancer therapy, prevention of aging, treatment of skin and hair diseases and improving skin and hairs well-being and appearance. It is thus of great interest to develop a specific approach that targets the skin stem cells by the use of a delivery system capable of overcoming the skin barrier.

Changing the cellular metabolic pattern and gene therapy are also possible ways for forward modulation and positive adjustment of stem cells activity. However, the various types of skin stem cells activity modulation are still in their infancy and there are plenty of obstacles to overcome to reach adequate effectiveness. Beside the problem of the skin barrier, one of the main concerns is the cell specificity and selectivity of the therapeutic intervention. Since the relationship between stem cells and skin problems is very important, we should set stem cells as targets, but there is still a paucity of study in these regards and thus it is quite difficult to envisage a general pattern.

Taking all into account, in our opinion, there is a huge need for systematic studies in this field to find out the best method and improve successful approaches to reach the cells. Moreover, innovative topical delivery systems should be specifically designed and research work should focus on the selective targeting of cutaneous stem cells [10,78]. We hope that our work will attract attention of researchers toward this challenging aspect of solving skin problems targeting skin stem cells.

## Figures and Tables

**Table 1:** Strategies to enhance skin penetration.

Strategies to Enhance Skin Penetration		
PHYSICAL ENHANCEMENT	Removal of stratum corneum	Microdermabrasion
		Thermal ablation
	Stratum corneum bypass	Iontophoresis
		Electroporation
		Sonophoresis
		Magnetophoresis
		Radiofrequency
		Pressure wave
		Microneedling
		Needleless injection
CHEMICAL ENHANCEMENT	Enzymes	
	Hydration of stratum corneum	
	Chemical enhancers	
DELIVERY SYSTEMS	Liposomes	
	Solid Lipid Nanoparticles	
	Nanostructured Lipid Carriers	
	Microemulsions and nanoemulsions	
	Nanocrystals	
	Cell penetrating peptides	
COMBINING DELIVERY APPROACHES		

## Aknowledgments

S.M. wishes to thank Ambrosialab (Ferrara, Italy) for the financial support. S.M., A.B. and P.G.B. thanks Elisa Durini, PhD, for kind technical assistance.
